# Association between ambient particulate matters and anhedonia among patients with depression

**DOI:** 10.1007/s11356-023-31474-9

**Published:** 2023-12-16

**Authors:** Tianqin Xie, Yu Zhang, Hui Kong, Lianzi Guan, Lei Zhang, Jiakuai Yu, Peng Zhu, Shuangshuang Ma, Dao-min Zhu

**Affiliations:** 1 The School of Mental Health and Psychological Sciences, Anhui Medical University, Hefei, 230032 China; 2 Department of Sleep Disorders, Affiliated Psychological Hospital of Anhui Medical University, Hefei, 230022 China; 3 Hefei Fourth People’s Hospital, Hefei, 230022 China; 4 Department of Maternal, Child & Adolescent Health, School of Public Health, Anhui Medical University, Hefei, 230032 China; 5 School of Nursing, Anhui Medical University, Hefei, 230032 China

**Keywords:** Air pollution exposure, PM2.5, PM10, Depression, Anhedonia

## Abstract

Recent studies have linked ambient air pollution to depression. Anhedonia is a core symptom of depression which severely impacts on prognosis. The present study aims to investigate the association of PM2.5 and PM10 exposure with anhedonia in depressed patients. A total of 538 patients with depression who were hospitalized at the Fourth People’s Hospital of Hefei between June 2017 and December 2021 were included. We estimated ambient particulate matters exposure, including PM2.5 and PM10, using a satellite-based spatiotemporal model at a resolution of 1 km^2^. The revised Physical Anhedonia Scale (RPAS) and the revised Social Anhedonia Scale (RSAS) were evaluated. The association of ambient particulate matters and anhedonia was examined using multiple linear regression models, adjusted for potential confounders. We observed that exposure to PM2.5 were significantly associated with increased RSAS score and RPAS score, with the major effect in the 12-month exposure window (*β* = 1.238; 95%CI, 0.353, 2.123) and 18-month exposure window (*β* = 1.888; 95%CI, 0.699, 3.078), respectively. Meanwhile, PM10 levels were also significantly associated with increased RSAS score and RPAS score, with the major effect in the 18-month exposure window (*β* = 1.220; 95%CI, 0.439, 2) and 3-month exposure window (*β* = 1.602; 95%CI, 0.062, 3.143), respectively. Subgroup analysis showed that both PM2.5 and PM10 were significantly associated with anhedonia in females, patients < 40 years old, low family income group, and those who had a higher educational level. Our study suggests that long-term PM2.5 and PM10 exposure are associated with more severe anhedonia in patients with depression. These associations were different in subgroup by age, gender, family income, and educational level.

**Supplementary information:**

The online version contains supplementary material available at 10.1007/s11356-023-31474-9.

## Introduction

Depression is a worldwide health problem that can lead to disability, functional decline, and an elevated risk of suicide (Friedrich 2017). Epidemiological evidence suggests that 4.4% of the global population suffers from depression, which is ranked as the single largest contributor to non-fatal health loss (7.5% of all years lived with disability [YLDs]) (World Health Organization 2018). In China, the annual prevalence of depression is 6.8%, and the lifetime prevalence is 3.6% (Huang et al. 2019). Anhedonia, a diminished motivation and sensitivity to pleasurable stimuli, is a core clinical symptom of depression (Berrios and Olivares 1995, American Psychiatry Organization 2013). Up to 70% of patients with depression experience significant anhedonia, which severely impairs not only their social functioning but also their cognitive abilities (McIntyre et al. 2016). Anhedonia also becomes a prominent residual symptom in some patients after clinical cure and even leads to refractory depression (Ely et al. 2021). Thus, the identification of modifiable risk factors for anhedonia is needed to provide potential prevention and control.

Air pollution contains many individual pollutants, including particulate matter (PM), gaseous pollutants, and metallic and organic compounds (Liu et al. 2023; Wang et al. 2023). PM is the most widespread health threat which may cause increased risk of mortality (Silva et al. 2013), cancer (Christiani 2021), respiratory disease (Jung et al. 2017), and cardiorespiratory diseases (Katsoulis et al. 2014). Recent studies show that air pollution (particulate matter) may interfere with the normal structure and function of the brain with long-lasting effects on mental health, particularly both depressive symptoms and depressive disorders (Fan et al. 2020; Rautio et al. 2018). A meta-analysis reported that long-term exposure to particulate matter exposure with an aerodynamic diameter < 2.5 µm (PM2.5) to ambient air pollution is associated with an augmented risk of depression (Braithwaite et al. 2019). Wang et al. (2020) found that exposure to PM2.5 increases depressive symptoms in older Chinese adults. Zhang et al. (2019) found a positive association between 12- and 60-month PM10 exposure and the developing depression. Lim et al. (2012) evaluated data from 537 elderly adults in Korea and reported that emotional symptoms were more likely to be associated with air pollution exposure than somatic and affective symptoms. Niedzwiecki et al. (2020) found that particulate matter exposure during pregnancy is positively associated with PPD and symptoms of anhedonia and depression at 6 months postpartum. However, the relationships between anhedonia and ambient particulate matters in depressed patients remain unclear.

In this cross-sectional study, we leverage a sample of participants with depression to examine the specific association of ambient particulate matter (PM2.5, PM10) exposure to anhedonia and to investigate the existence of susceptible subgroups.

## Materials and methods

### Study design and participants

Five hundred sixty-two patients were recruited from Hefei Fourth People’s Hospital, Hefei, China, in 2017 to 2021. All patients were assessed by the Mini-International Neuropsychiatric Interview (MINI) 6.0.0 to confirm the diagnosis of major depressive disorders (MDD). Structured questionnaires were administered by all participants and were used to record sociodemographic characteristics, lifestyle, and depressive symptoms. Ground PM2.5 and PM10 concentration and satellite AOD data were obtained from 2017 to 2021 according to each participant’s residential address.

Participants were enrolled as the following criteria: (1) diagnosis of MDD according to Diagnosis and Statistical Manual-5th edition (DSM-V), (2) Han Chinese ethnicity, (3) age 18–65 years, (4) be able to cooperate in completing questionnaires. The exclusion criteria were as follows: (1) other concurrent psychiatric disorders defined in the DSM-V such as schizophrenia and substance use disorders, (2) and current severe physical conditions (e.g., neurological diseases, malignancy, cardiovascular diseases, respiratory diseases, severe trauma, state of pregnancy, or breastfeeding), (3) moved in the last 2 years. After the strict clinical screening, we excluded 40 depressed patients, among whom 18 patients missed information on anhedonia and address, 12 patients moved in the last 2 years, and 8 patients diagnosed with cardiovascular diseases or respiratory diseases. The final analyses included 538 depressed patients.

All subjects received and signed informed consent forms prior to enrollment, and this study was approved by the Ethics Committee of Anhui Mental Health Center (AMHC).

### Exposure measurements

Exposure to PM2.5 and PM10 was estimated at each participant’s address, which was reported in the questionnaire coordinates and geocoded into latitude and longitude data. A satellite-based spatiotemporal model with a high spatial resolution of 1 × 1 km on the basis of National Aeronautics and Space Administration (NASA) aerosol optical thickness (AOD) data were used to estimate ambient PM2.5 and PM10 concentrations. And we used 1 month, 3 months, 6 months, 12 months, 18 months, and 24 months before the survey day as exposure windows.

### Clinical assessments

#### Revised Social Anhedonia Scale (RSAS)

Social anhedonia is defined as an impaired ability to feel pleasure in the interpersonal domain, and the Chinese version of the revised social anhedonia scale is a 40-item self-assessment questionnaire with a total score of 0 to 40, with higher scores indicating a more severe social anhedonia. The items are scored relative to standard answers. Items requiring reverse scoring are “False,” and items not requiring reverse scoring are “True” based on the standard answer. An item is scored as a “1” if the response to the item is consistent with the standard answer for that item; otherwise, it is scored as a “0.” The total score is the sum of the scores for each item. A series of studies have shown that it has a high excellent coefficient (Hu et al. 2018).

#### Revised Physical Anhedonia Scale (RPAS)

The RPAS is a 61-item self-rated questionnaire to assess whether participants can experience physical satisfaction from typical pleasurable stimuli such as food and situations, and each item is scored in the same way as the RSAS. The total score is the sum of the scores for each item. This psychometric scale provides a reliable and valid measure for patients with depression (Kollias et al. 2008).

#### Hamilton Depression Scale (HAMD_24_)

This is the most commonly used scale worldwide to assess the severity of depression with high reliability and validity. The HAMD_24_ has 24 items. Most of the HAMD_24_ items (item 1, 2, 3, 7, 8, 9, 10, 11, 15, 19, 20, 22, 23, 24) are scored on a scale of 0–4 according to the severity of each symptom. The total score is the sum of the scores for each item. Other items are scored on a scale of 0–2. Score higher than 8 in HAMD_24_ indicates the presence of depressive symptoms (Hamilton 1967).

#### Hamilton Anxiety Rating Scale (HAMA)

HAMA consists of 14 entries and two factor categories: somatic anxiety (7, 8, 9, 10, 11, 12, and 13 entries) and psychiatric anxiety (1, 2, 3, 4, 5, 6, and 14 entries), depending on the severity of each symptom using a 0 to 4 scoring method. The total score is the sum of the scores for each item. Score higher than 7 indicates the presence of anxiety symptoms. The scale was developed by Hamilton in 1959 and has been widely used to assess anxiety symptoms with good validity and reliability (Zimmerman et al. 2017).

### Covariates

In addition to clinical assessments, we controlled for potential confounding covariates in the analysis. The following covariates were included in data analysis: age (years), gender (male and female), educational level (middle school or below, high school or above), smoking status (non-smoker, smoker), alcohol drinking (non-drinker, drinker), the frequency of physical activity in the past 1 month (sedentary, 1–2 times/week, 3–5 time/week, > 6 time/week), use of antidepressants in the past 1 month (“yes,” “no”), season of visit, and family income (< 4000 RMB, ≥ 4000 RMB). Body mass index (BMI) was calculated as weight in kilograms divided by squared height in meters.

### Statistical analysis

Continuous variables were presented as mean ± standard deviation if distributed normally, or expressed as medians (interquartile ranges) if distributed skewed. Categorical variables were presented as frequency (percentage). And we used multiple linear regression models to investigate the association between PM2.5 and PM10 on anhedonia among all study participants. The measure of variance inflation factors (VIFs < 2) was used to avoid the multicollinearity among the variables (Yu et al. 2022). Model 1 adjusted for age, sex, educational level, family income, and employment; Model 2 further adjusted for BMI, smoking status, drinking status, and physical activity; Model 3 further adjusted for season of visit and use of antidepressants. All results were expressed as changes in RSAS and RPAS score associated with an IQR increase in exposure and corresponding 95% confidence intervals (CI). To analyze the effect of different ambient particulate matters exposure windows, we calculated 1-month, 3-month, 6-month, 12-month, 18-month, and 24-month average levels. Subgroup analyses were conducted stratified by gender, age, and family income. All analyses were performed using the R software (version 3.6.0, R Foundation for Statistical Computing).

## Result

### General characteristics

Table 1 shows characteristics of the study population. The mean age of the study population was 41.2 years (standard deviation [SD] = 13.1) with 68% females. 43.5% of the study population had high school or above educational status, and the mean BMI is 22.7 kg/m^2^ (SD = 3.2). As for the smoking and drinking status, 15.2% and 9.8% of participants were smokers and alcohol drinkers. Fig. S1 showed the distribution of 538 participants in 16 cities from Anhui province of China. One-month, 3-month, 6-month, 12-month, 18-month, and 24-month average concentrations with the median and IQR of air pollution among study participants are shown in Table S1.

**Table 1:** Descriptive characteristics of the study population (*N* = 538)

Variable	Mean ± SD (median, range)
Age, years	41.2 ± 13.1
Gender, *n* (%)	
Male	172 (32%)
Female	366 (68%)
Education level, *n* (%)	
Middle school or below	304 (56.5%)
High school or above	234 (43.5%)
Family income, *n* (%)	
< 4000RMB	252 (46.8%)
≥ 4000RMB	286 (53.2%)
Employment, *n* (%)	
Employed	330 (61.3%)
Unemployed, home maker, or other	208 (38.7%)
Smoking status, *n* (%)	
Non-smoker	456 (84.8%)
Smoker	82 (15.2%)
Drinking status, *n* (%)	
Non-drinker	485 (90.2%)
Drinker	35 (6.5%)
Physical activity, *n* (%)	
Sedentary	227 (42.2%)
1–2 times/week	120 (22.3%)
3–5 time/week	101 (18.8%)
> 6 time/week	90 (16.7%)
BMI (kg/m^2^)	22.7 ± 3.2
Use of antidepressants, n (%)	
Yes	401 (75.1%)
No	134 (24.9%)
Season of visit	
Winter/spring	270 (50.2%)
Summer/autumn	268 (49.8%)
Scale score	
HAMD-24	29.8 ± 8.9
HAMA	31.6 ± 6.7
RSAS	22.6 ± 5.6
RPAS	33.9 ± 8.9

*SD* standard deviation, *BMI* body mass index, *HAMD-24* Hamilton Depression Scale-24, *HAMA* Hamilton Anxiety Rating Scale, *RSAS* Revised Social Anhedonia Scale, *RPAS* Revised Physical Anhedonia Scale

### Associations between ambient particulate matters and anhedonia

**Fig. 1:**
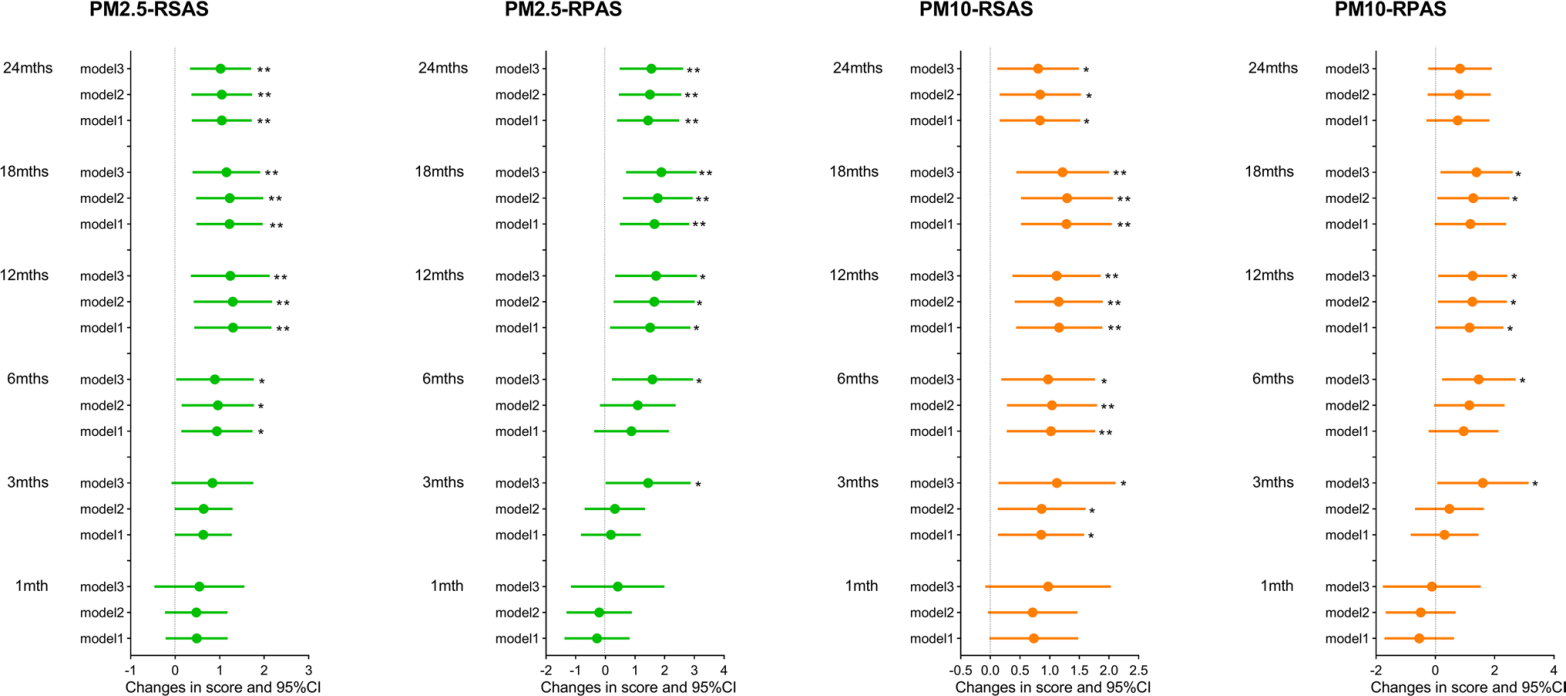
Adjusted change (95% confidence interval (CI)) of RSAS and RPAS scores for an interquartile range (IQR) change in ambient particulate matters (*N* = 538). Notes: *p < 0.05, **p < 0.01. Abbreviations: CI, confidence interval; IQR, interquartile range; PM2.5, particulate matter with aerodynamic diameter ≤ 2.5 µm; PM10, particulate matter with aerodynamic diameter ≤ 10 µm. Model 1 is adjusted for age, sex, educational level, family income, and employment. Model 2 further adjusted BMI, smoking status, drinking status, and physical activity on the basis of Model 1. Model 3 further adjusted season of visit and use of antidepressants on the basis of Model 2

Figure 1 shows the adjusted associations between different exposures to ambient particulate matters. We observed that PM2.5 levels for the 6-month, 12-month, 18-month, and 24-month exposure windows were significantly associated with RSAS score in Model 3, with the major effect in the 12-month exposure window (*β* = 1.238; 95%CI, 0.353, 2.123). As for PM2.5 and RPAS score, PM2.5 exposure for the 3-month, 6-month, 12-month, 18-month, and 24-month windows were significantly associated with RPAS score in fully adjusted model, with the major effect in the 18-month exposure window (*β* = 1.888; 95%CI, 0.699, 3.078). For PM10 and RSAS score, the significant exposure windows were the 3 months, 6 months, 12 months, 18 months, and 24 months, with the major effect in the 18-month exposure window (*β* = 1.220; 95%CI, 0.439, 2). And in regard to PM10 and RPAS score, after fully adjusted, IQR increases in PM10 levels for the 3-month (*β* = 1.602; 95%CI, 0.062, 3.143), 6-month (*β* = 1.466; 95%CI, 0.288, 2.704), 12-month (*β* = 1.218; 95%CI, 0.120, 2.442), and 18-month (*β* = 1.390; 95%CI, 0.172, 2.608) exposure windows were, respectively, associated with RPAS score.

### Subgroup analyses

**Fig. 2:**
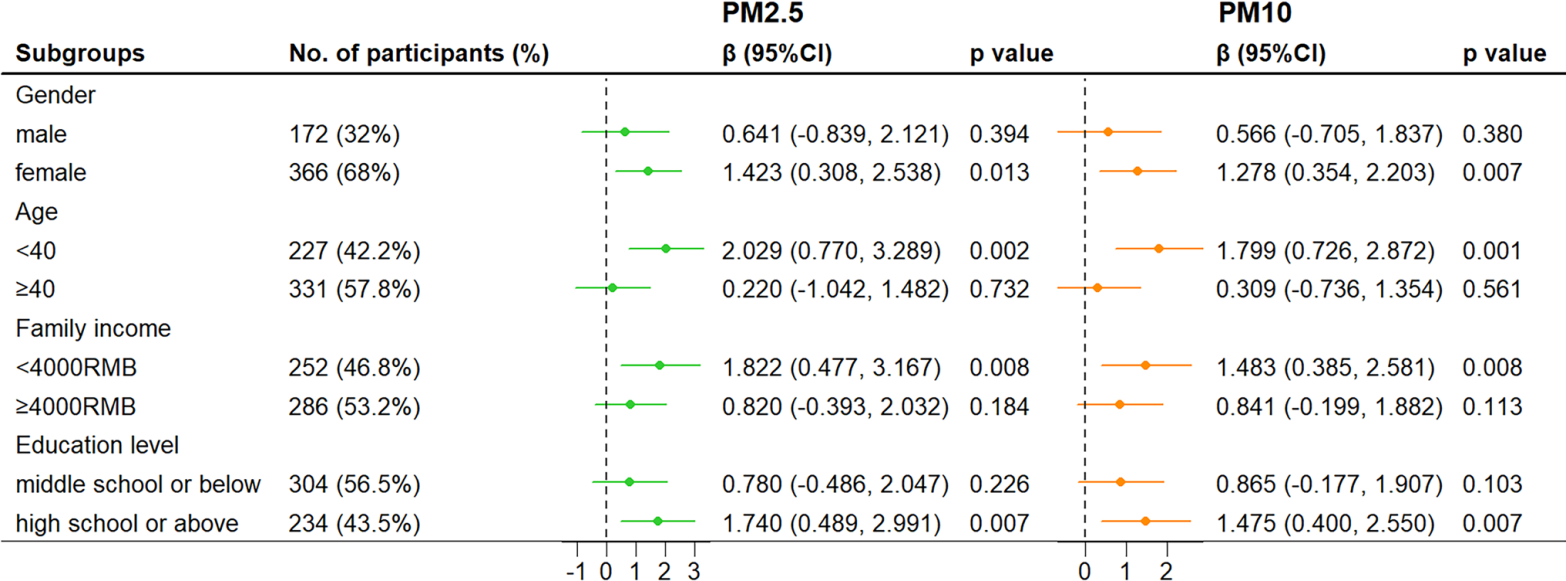
Subgroup analysis for the association between 12-month exposure to ambient particulate matters and RSAS scores. Notes: All results were adjusted for age, sex, educational level, family income, employment, BMI, smoking status, drinking status, physical activity, season of visit, and use of antidepressants. Notes: *p < 0.05, **p < 0.01. Abbreviations: CI, confidence interval; PM2.5, particulate matter with aerodynamic diameter ≤ 2.5 µm; PM10, particulate matter with aerodynamic diameter ≤ 10 µm

**Fig. 3:**
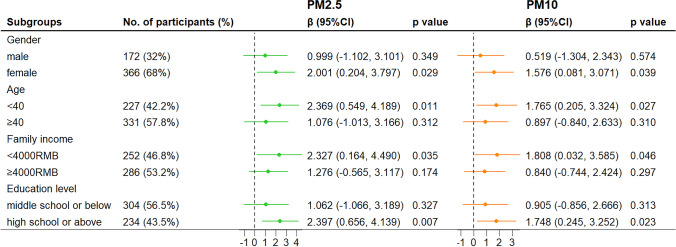
Subgroup analysis for the association between 12-month exposure to ambient particulate matters and RPAS scores. Notes: All results were adjusted for age, sex, educational level, family income, employment, BMI, smoking status, drinking status, physical activity, season of visit, and use of antidepressants. Notes: *p < 0.05, **p < 0.01. Abbreviations: CI, confidence interval; PM2.5, particulate matter with aerodynamic diameter ≤ 2.5 µm; PM10, particulate matter with aerodynamic diameter ≤ 10 µm

In subgroup analysis, the effect size of exposure to PM2.5 and PM10 on RSAS and RPAS score was significantly higher among those who were female or younger than 40 years old. We also found the association between ambient particulate matters and anhedonia would be stronger in participants who had a lower family income and a higher educational level (Figs. 2 and 3).

## Discussion

In the present study, we found that RSAS and RPAS score was associated with increased exposure to ambient particulate matters such as PM2.5 and PM10 in depressed patients after adjusting for potential confounders. Stronger associations were estimated among female, patients < 40 years old, low family income group, and those who had a higher educational level. Our results indicate that anhedonia in depressed patients was positively associated with PM2.5 and PM10 exposure, and female, patients < 40 years old, low family income group, and those who had a higher educational level were more susceptible to PM exposure.

This study initially found a positive association between environmental particulate matter and anhedonia in patients with depression. Despite few studies on ambient particulate matters exposure and anhedonia, two studies conducted among pregnant women showed similar results. Results from a birth cohort in Mexico City (509 mothers with available data) suggest that particulate matter exposure during pregnancy is positively associated with PPD and symptoms of anhedonia and depression at 6 months postpartum (Niedzwiecki et al. 2020). Another study from the USA found that increased PM2.5 exposure in mid-pregnancy was associated with increased depressive and anhedonia symptoms (Sheffield et al. 2018). Mechanistic data indicates that inflammation may be a pathophysiologic pathway to anhedonia. Felger et al. (2017) found that inflammatory stimuli reduce neural activity and dopamine release in reward-related brain regions in association with reduced motivation and anhedonia. And increasing evidence suggests that inflammation may mediate the relationship between air pollution and anhedonia. Hogan et al. (2015) reported that PM2.5 exposure could trigger depression-like responses (anhedonia) in mice through upregulates neuroinflammatory cytokines and altering structural changes in the CA1 hippocampus. Future studies are needed to further explore the potential mechanism linking air pollution and anhedonia. However, results from MOBILIZE Boston Study found no evidence of a positive association between long-term exposure to PM2.5 and depressive symptoms using the Revised Center for Epidemiological Studies Depression Scale (CESD-R) in older adults (Wang et al. 2014). One possible reason is that CESD-R items do not measure the diagnostic criteria of anhedonia (Smarr and Keefer 2011). A study from the French CONSTANCES cohort suggests that the relationship between air pollution and depressive symptoms is not fully explained by somatic symptoms (Zare Sakhvidi et al. 2022). Additional studies about air pollution and different dimensions of depressive symptoms in human cohorts are warranted.

Notably, the present study found that longer-term cumulative air pollution exposure was significantly associated with anhedonia, but the result was not found in the short term. Similar results were found in a Scottish study assessing the relationship between air pollution and mortality, in which Beverland et al. (2012) compared associations between short-term exposure to black smoke (BS) and mortality with long-term exposure-mortality associations in two cohorts and the results showed that short-term exposure-mortality associations were substantially lower than equivalent long-term associations.. The difference in the magnitude of the effects between long-term and short-term exposures may be due to the greater and more persistent cumulative effects of long-term exposures.

In subgroup analysis, interestingly, we observe stronger associations between anhedonia and PM exposure among female, patients < 40 years old, low family income group, and those who had a higher educational level. Previous studies have shown that particulate matter exposure risks are higher for women than for men (Bell et al. 2013), which may be attributed to differences in life stage, co-exposures, hormonal status, or other factors (Clougherty 2011). As in our study, higher susceptibility of individuals younger than 40 years old of ambient air pollution exposure on anhedonia has been reported. A recent study from Canada found that the negative effect of air pollution on depressed mood may be greater in younger people (Szyszkowicz and Rowe 2014), and this discrepancy may result from different indoor/outdoor activity patterns and occupational exposures. The impact of education on particulate matter-associated risks is controversial. In our study, subgroup analyses showed few significant associations in participants with low education. Filleul et al. (2004) found greater associations between particles and mortality among the more highly educated. But Martins et al. (2004) reported that the effect of particulate matters was negatively correlated with percentage of people with college education. One possible explanation of our finding is that residential groups by educational level may lead to differences in exposure to particulate matter of different source and component. In addition, our results suggested that the relationship between ambient particulate matters and anhedonia was more significant in the low-income groups were all higher than in high-income groups. This finding is consistent with the finding of a recent meta-analysis (Bell et al. 2013), which may be due to the difference in personal self-protection strategies against air pollution (Carlsten et al. 2020).

Our study had several strengths. First, to the best of our knowledge, this is the first study to investigate associations between anhedonia among depressed patients and air pollution after controlling for individual effects. Most of our patients were in severe depression; the diagnoses were based on semi-structured interview kit. Second, PM2.5 and PM10 exposure were assessed accurately using high-performance satellite-based technology with a high spatial resolution of 1 km^2^. We also acknowledge some limitations. First, the sample size in this clinical research is relatively smaller; future studies should target on larger-sized cohorts of depressive patients. Second, anhedonia was assessed using only the RSAS and RPAS. The stages of reward processing were not assessed (e.g., motivation, consumption-based anhedonia). Third, other confounding factors that may affect the estimation effect such as traffic noise and surrounding green space have not been considered, but it remains unknown to what extent these factors would affect our findings. Future studies could further explore the association between other environmental exposures and anhedonia. Lastly, we did not measure levels of indoor air pollution. Levels of PM2.5 and PM10 were estimated at the home addresses of the participants. Although we took into account the confounding factor of smoking which is one of the important components of indoor PM2.5 sources, it may not accurately reflect actual personal exposure. Future studies may better use personal exposure data for health-related exploration.

## Conclusion

This is the first report to investigate the relationship between anhedonia among depressive patients and air pollution. The results of this study showed that exposure to PM2.5 and PM10 was significantly associated with anhedonia in patients with depressive disorders. In addition, we found that female, younger people (< 40 years old), and those with lower family income and higher educational level were more vulnerable to PM2.5 and PM10. This study provides clinical evidence to elucidate the etiological pathway of air pollution on depressive orders and also reference for the public health sector in the prevention and treatment of refractory depression.

### Supplementary information

Below is the link to the electronic supplementary material.

Supplementary file1 (DOCX 15 KB)

Supplementary file2 (TIF 70862 KB)

## Data Availability

The data sets used and analyzed during the current study are available from the corresponding author on reasonable request.
