# Supplementary material for: Association between ambient particulate matters and anhedonia among patients with depression
Source: Environ Sci Pollut Res Int. 2023 Dec 16;31(3):4539–46. doi: 10.1007/s11356-023-31474-9 (PMC10794277; doi:10.1007/s11356-023-31474-9)
Supplement: Supplementary file 1 — Supplementary file1 (DOCX 15 KB) [file 11356_2023_31474_MOESM1_ESM.docx]

**Supplementary**

**Table S1**

1-month, 3-month, 6-month, 12-month, 18-month and 24-month average concentrations of ambient air pollutants among study participants (*N*=538).

| Variable | Median | IQR |
| --- | --- | --- |
| PM2.5 (1 month) (ug/m^3^) | 42.0 | 29.9-58.8 |
| PM2.5 (3 month) (ug/m^3^) | 42.1 | 30.9-56.5 |
| PM2.5 (6 month) (ug/m^3^) | 44.6 | 33.1-59.2 |
| PM2.5 (12 month) (ug/m^3^) | 48.1 | 37.9-54.6 |
| PM2.5 (18 month) (ug/m^3^) | 51.2 | 39.4-65.5 |
| PM2.5 (24 month) (ug/m^3^) | 51.3 | 42.8-63.0 |
| PM10 (1 month) (ug/m^3^) | 76.8 | 56.3-97.5 |
| PM10 (3 month) (ug/m^3^) | 77.1 | 57.3-95.2 |
| PM10 (6 month) (ug/m^3^) | 76.7 | 61.4-93.3 |
| PM10 (12 month) (ug/m^3^) | 79.9 | 67.2-88.7 |
| PM10 (18 month) (ug/m^3^) | 78.8 | 66.2-87.7 |
| PM10 (24 month) (ug/m^3^) | 78.3 | 67.4-85.2 |

Abbreviation: Note. IQR, interquartile range; PM2.5, particulate matter with an aerodynamic diameter of ≤2.5 μm; PM10, particulate matter with an aerodynamic diameter of ≤10 μm.

**Fig. S1.** Sample distribution.
